# The WNT/β-catenin signaling inhibitor XAV939 enhances the elimination of LNCaP and PC-3 prostate cancer cells by prostate cancer patient lymphocytes *in vitro*

**DOI:** 10.1038/s41598-019-41182-5

**Published:** 2019-03-18

**Authors:** Dmitry Stakheev, Pavla Taborska, Zuzana Strizova, Michal Podrazil, Jirina Bartunkova, Daniel Smrz

**Affiliations:** Institute of Immunology, Charles University, 2nd Faculty of Medicine and Motol University Hospital, Prague, Czech Republic

## Abstract

Upregulated Wnt/β-catenin signaling is associated with increased cancer cell resistance and cancer cell-elicited immunosuppression. In non-neoplastic immune cells, upregulated Wnt/β-catenin is, however, associated with either immunosuppression or immunostimulation. Therefore, it is difficult to predict the therapeutic impact inhibitors of Wnt/β-catenin signaling will have when combined with cancer immunotherapy. Here, we evaluated the benefit(s) of the Wnt/β-catenin signaling inhibitor XAV939 in the *in vitro* elimination of LNCaP prostate cancer cells when cocultured with lymphocytes from patients with localized biochemically recurrent prostate cancer (BRPCa). We found that 5 µM XAV939 inhibited β-catenin translocation to the nucleus in LNCaP cells and CD4^+^ BRPCa lymphocytes without affecting their proliferation and viability. Preconditioning BRPCa lymphocytes with 5 µM XAV939 accelerated the elimination of LNCaP cells during the coculturing. However, during subsequent re-coculturing with fresh LNCaP cells, BRPCa lymphocytes were no longer able to eliminate LNCaP cells unless coculturing and re-coculturing were performed in the presence of 5 µM XAV939. Comparable results were obtained for PC-3 prostate cancer cells. These findings provide a rationale for combining cell-based immunotherapy of PCa with inhibitors of Wnt/β-catenin signaling.

## Introduction

Wnt/β-catenin signaling is an evolutionarily conserved pathway that is involved in many biological processes, such as embryogenesis, tissue homeostasis, cell development, proliferation, survival and differentiation^1^. The central effector of Wnt/β-catenin signaling is β-catenin^2^. β-catenin has a large number of binding partners that regulate its transcription and allow its crosstalk with other signaling pathways^3^. In the absence of activated Wnt signaling, β-catenin is degraded, which ensures the maintenance of low levels of β-catenin in the cytosol. When Wnt/β-catenin signaling is activated, β-catenin accumulates in the cytosol and translocates to the nucleus, where it interacts with transcription factors^2^.

Wnt/β-catenin signaling is often upregulated in cancer cells, which confers cells a stem-like phenotype that increases the cancer cell self-renewal capacity, multi-differential potential, and features of epithelial-to-mesenchymal transition^3–5^. The consequence is that cancer cells with upregulated Wnt/β-catenin signaling are often associated with more aggressive disease^6^, metastases^7,8^, and increased resistance to hormonal therapy^9^, chemotherapy^10^, or radiotherapy^11^. Upregulated Wnt/β-catenin signaling in cancer cells is also responsible for cancer cell-elicited immunosuppression^12,13^. Therefore, Wnt/β-catenin signaling has become an attractive target for the treatment of multiple cancers. Currently, there are several ongoing clinical trials of small molecule inhibitors targeting the activity of Wnt/β-catenin signaling components^14,15^. One group of Wnt/β-catenin signaling inhibitors is tankyrase inhibitors^16^, which block the accumulation of β-catenin in the cytosol^17^.

Although inhibition of Wnt/β-catenin signaling seems to be a promising cancer treatment option, the impact that such inhibition will have on the immune system under a specific disease condition is difficult to predict because inhibition of Wnt/β-catenin signaling can have different effects on the regulation of different indices of immune responses^18,19^. Therefore, even though Wnt/β-catenin signaling inhibitors have been found to be effective in cancer treatment in combination with other treatment modalities^20,21^, their performance in combination with immunotherapy still remains largely unpredictable. It is particularly important to evaluate this response under specific disease conditions when these inhibitors are administered in combination with immunotherapy, in which immune cell-mediated elimination of cancer cells is the key mechanism that delivers the therapeutic impact.

To evaluate how inhibition of Wnt/β-catenin signaling in either cancer cells or immune cells or both may affect the elimination of prostate cancer (PCa) cells by PCa patient’s lymphocytes under a specific disease condition, we used an *in vitro* culture system. This system consisted of the fluorescent TagFP635-transfected Lymph Node Carcinoma of the Prostate (LNCaP) cancer cell line (TagFP635-LNCaP), peripheral blood-isolated lymphocytes from patients with localized biochemically recurrent PCa (BRPCa lymphocytes), and the tankyrase inhibitor XAV939. In this system, we used a concentration of XAV939 that we found did not compromise viability, proliferation, and differentiation of LNCaP cells and BRPCa lymphocytes but was still able to inhibit β-catenin translocation to the nucleus in cancer cells and a subset of BRPCa lymphocytes. Cancer cell elimination was evaluated for an extended period of time in a 5-day coculture of BRPCa lymphocytes with TagFP635-LNCaP cells and a follow-up 10-day re-coculture with fresh TagFP635-LNCaP cells during which the number of TagFP635-LNCaP cells was monitored through their fluorescence. The key findings of the study were reproduced with another prostate cancer cell line, PC-3.

## Materials and Methods

### Preparation of TagFP635-LNCaP and TagFP635-PC-3 cells

The LNCaP^22^ and PC-3^23^ cell lines were obtained from American Type Culture Collection (ATCC, Manassas, VA). LNCaP cells (30–50 × 10^3^ cells) in 2 ml of fetal bovine serum-containing culture medium [RPMI 1640 medium with 10% fetal bovine serum (HyClone, GE Healthcare Life Sciences, South Logan, UT), 100 U/ml penicillin-streptomycin, 2 mM Glutamax] were seeded in a flat-bottom 6-well plate and cultured at 37 °C in 5% CO_2_ for 2 days. PC-3 cells (10 × 10^3^ cells) in 1 ml of the fetal bovine serum-containing culture medium were seeded in a flat-bottom 12-well plate and cultured in the same way as LNCaP cells for 2 days. The LNCaP cell culture was supplemented with MISSION pLKO.1-puro-CMV-SHC013V TagFP635 lentiviral particles (Sigma-Aldrich, St. Louis, MO, SHC013V). The PC-3 cell culture was supplemented with FP635 Lentivirus (pLVX-Puro) (Applied Biological Materials, Inc., Richmond, BC, Canada, LVP010023). Virus-supplemented cells were cultured at 37 °C in 5% CO_2_. After 1 day, the lentivirus-containing supernatant was removed from LNCaP cells, new culture medium was added, and the cells were cultured for 2 additional days. The PC-3 culture was left intact until day 3 post virus supplementation. The supernatant was removed from both LNCaP and PC-3 cells, and the cells were cultured in the presence of 1 µg/ml (LNCaP) or 5 µg/ml (PC-3) of puromycin (Sigma) for 6 days, the medium with puromycin was replaced every 2^nd^ day. The cells were then transferred to T75 flasks and cultured in the presence of puromycin for 7 days, and the medium with puromycin was replaced every 2^nd^–3^rd^ day. Lentiviral particle-transduced and puromycin-selected LNCaP and PC-3 cells were then cryopreserved. After reconstitution in culture media, the selected cells were over 90% positive for TagFP635 fluorescence, and this positivity was stable during the following culturing.

### Patients’ samples

BRPCa patients’ lymphocytes were prepared as described previously^24^. Cryopreserved lymphocytes of 12 BRPCa patients after radical prostatectomy or salvage radiotherapy were available for this study. Patients’ median age was 64 years (age range 55–74 years), the median Gleason score was 6 (range 5–7) and PSA was 0.146 ng/ml (range 0.045–0.468 ng/ml). The experiments in this study were approved by the appropriate institutional and/or national research ethics committee – the Ethics Committee of the Motol University Hospital in Prague. Written informed consent was approved by the Ethics Committee of the Motol University Hospital in Prague and was obtained from all patients before the study procedures.

### Lymphocyte proliferation assay

BRPCa lymphocytes were prepared in flat-bottom 48-well plates as above. The cells were then stimulated by supplementation of the wells with 5 µl of 1 mg/ml phytohaemagglutinin (PHA) (Sigma). The stimulated cells were then cultured at 37 °C in 5% CO_2_. After 3 days, the cell number was determined, the cell suspension was transferred to 15 ml conical tubes (TPP, Trasadingen, Switzerland) and the cells were pelleted by centrifugation at 240 × g for 10 min at room temperature. The supernatant was removed, and the cells were resuspended in 200 µl cold PBS/EDTA and transferred to 96 V-bottom wells (Nalgene, Rochester, NY). The cells were pelleted by centrifugation at 280 × g for 5 min at 4 °C, washed with 200 µl cold PBS/EDTA and stained for 30 min at 4 °C with LIVE/DEAD Fixable Aqua Stain (Thermo Scientific). The cells were washed with cold PBS/EDTA and, using a fixation/permeabilization set (eBiosciences), fixed for 30 min at 4 °C, pelleted by centrifugation at 600 × g for 5 min at 4 °C and permeabilized overnight at 4 °C with the permeabilization solution supplemented with 50 × diluted normal mouse serum (Thermo Scientific). The cells were pelleted and stained in the mouse serum-containing permeabilization solution containing the following fluorophore-conjugated protein-specific antibodies: CD4-PE-Cy7 (eBiosciences, San Diego, CA), CD8-Alexa Fluor 488 and Ki67-PE (Exbio, Prague, Czech Republic) for 30–60 min at 4 °C. After washing with cold PBS/EDTA, the stained cells were analyzed by FACSAria II (Becton Dickinson, Heidelberg, Germany) and FlowJo software (TreeStar, Ashland, OR).

### β-catenin translocation analysis

Cryopreserved BRPCa lymphocytes were reconstituted in a human serum containing culture medium [LM; RPMI 1640 medium (Thermo Scientific) with 5% pooled human serum (One Lambda, Kittridge, CA), 100 U/ml penicillin-streptomycin, 2 mM Glutamax, 1 mM sodium pyruvate and a non-essential amino acid mix (Thermo Scientific)] as described previously^24^. The reconstituted cells were resuspended in LM at a concentration of 1.0 × 10^6^ cells/ml, and 1 ml of the cell suspension was transferred to a flat-bottom 48-well plate well. The cell suspension was supplemented with XAV939 and cultured at 37 °C in 5% CO_2_ for 2 days. The cell suspension was recovered and transferred to 15 ml conical tubes (TPP), and the cells were pelleted by centrifugation at 240 × g for 10 min at room temperature. The supernatant was removed, and the cells were resuspended in 200 µl cold PBS (4 °C) with 2 mM EDTA (PBS/EDTA) and then transferred to 96 V-bottom wells (Nalgene). The cells were pelleted, washed with 200 µl cold PBS/EDTA, stained with LIVE/DEAD Fixable Aqua Stain (Thermo Scientific, Waltham, MA), fixed and permeabilized as above. The cells were pelleted and stained in the mouse serum-containing permeabilization solution with the following fluorophore-conjugated protein-specific antibodies: CD4-PE-Cy7 (eBiosciences), CD8-Alexa Fluor 488 (Exbio, Prague, Czech Republic), β-catenin-FITC (Miltenyi Biotec, Gladbach, Germany) and a DNA stain DRAQ5 (5 µM, Thermo Scientific) for 30–60 min at 4 °C. The stained cells were washed twice with cold PBS/EDTA and then analyzed with a high-speed imaging flow cytometer, ImageStreamX MKII (Amnis Corporation, Seattle, WA). Bright field and fluorescent images were collected at 40 x magnification, and 1.0 × 10^5^ gated single cells were acquired for each sample. The IDEAS analysis software (Amnis Corporation) was used to determine β-catenin nuclear localization. Nuclear localization was determined by the Pearson correlation coefficient as previously described by^25^.

### Immunoblotting

Immunoblotting was performed on LNCaP lysates prepared and analyzed as described previously^26^ with the exception that the reducing agent, 20% (v/v) 2-mercaptoethanol, was replaced with 10% (w/v) DL-Dithiothreitol (Sigma). The chemiluminescence from immunoblots was visualized using an imaging camera, ALLIANCE 9.7 CHROMA R/G/B UVIpure (UVITEC, Cambridge, UK). The images acquired within a linear range of saturation were densitometrically analyzed with Quantity One software (Bio-Rad, Hercules, CA).

### Cell culture and XAV939 treatment

Cryopreserved BRPCa lymphocytes were reconstituted as above and resuspended in LM with 20 IU/ml IL-2 (PeproTech, Rocky Hill, NJ) at 0.5–2.0 × 10^6^ cells/ml. The cell suspension was then supplemented with XAV939 and cultured at 37 °C in 5% CO_2_ for 2 days. The cells were harvested, transferred to 15 ml conical tubes (TPP), centrifuged at 240 × g for 10 min at room temperature, and resuspended in LM with 20 IU/ml IL-2 at a concentration of 1 × 10^6^ cells/ml. TagFP635-LNCaP (250 × 10^3^) or TagFP635-PC-3 (50 × 10^3^) cells in 1 ml of KM or KM supplemented with XAV939 were seeded in a flat-bottom 48-well plate well (Nalgene) and cultured for 2 days at 37 °C in 5% CO_2_. Next, the supernatant was removed, and 2-day XAV939-treated BRPCa lymphocytes (1 × 10^6^ cells) were added to XAV939-treated TagFP635-cancer cells. The lymphocytes were allowed to sediment for 10 min, and the mean fluorescence intensity (MFI) of TagFP635-cancer cells in the wells was determined. The cells were then cocultured for 5 days at 37 °C in 5% CO_2,_ and the MFI of TagFP635-cancer cells in the coculture was determined on days 3 and 5. At day 5, the cocultured cells were resuspended, and the whole cell suspension was transferred to a new flat-bottom 48-well plate well (Nalgene) with fresh TagFP635-cancer cells prepared as described above. The MFI of TagFP635-cancer cells in the new coculture (re-coculture) was determined after 10 min of sedimentation, and the cells were re-cocultured for 10 days at 37 °C in 5% CO_2_. On days 2, 5, 7 and 10, the MFI of TagFP635-cancer cells in the re-coculture was determined. Alternatively, BRPCa lymphocytes and TagFP635-cancer cells that were not pre-treated with XAV939 were cocultured and re-cocultured in the presence of XAV939.

### Fluorescent microscopy and image analysis

TagFP635-LNCaP or TagFP635-PC-3 cells were imaged using Olympus IX81 microscope in the RFP channel under the same acquisition settings during the entire experiment. The microscope objective lens was a UPLSAPO 10 × (numerical aperture, 0.4). For each sample in the 48-well plate well, 5–6 randomly acquired images of TagFP635-cancer cells were obtained. Each image covered an area of 9.61 mm^2^. The MFI of RFP in the acquired images was calculated as the mean gray value using ImageJ software (NIH).

### T lymphocyte phenotype

The cells were stained with fluorophore-conjugated protein-specific antibodies as described previously^27^. The antibodies were CD8-PE Dy594, CD4-Pacific Blue, CD45RO-PE, CD3-Alexa Fluor 700 (Exbio), and CCR7-PerCP Cy5.5 (BioLegend, San Diego, CA). The stained cells were pelleted, washed twice with cold PBS/EDTA, and after supplementation with DAPI (100 ng/ml; Thermo Scientific, Waltham, MA), immediately analyzed by FACSAria II and FlowJo software as above. The population of live cells was gated as DAPI-negative cells, and the T lymphocyte population was gated as CD3^+^ cells. The gated CD4^+^ or CD8^+^ T lymphocytes with the phenotype of central memory (CM) were CD45RO^+^CCR7^+^; effector memory: CD45RO^+^CCR7^−^; naïve: CD45RO^−^CCR7^+^; and terminally differentiated (TEMRA): CD45RO^−^CCR7^−^.

### Statistical analysis

Statistical significance was calculated from the indicated sample size (*n*) by GraphPad Prism (GraphPad software, La Jolla, CA) and determined by the indicated test. P < 0.05 was considered significant.

### Ethical approval

The studies were approved by the appropriate institutional and/or national research ethics committee (the Ethics Committee of the Motol University Hospital in Prague) and were performed in accordance with the ethical standards as laid down in the 1964 Declaration of Helsinki and its later amendments or comparable ethical standards.

### Informed consent

Informed consent was obtained from all individual participants included in the study.

## Results

### XAV939 (5 µM) does not impact the expansion of LNCaP cells

**Figure 1:**
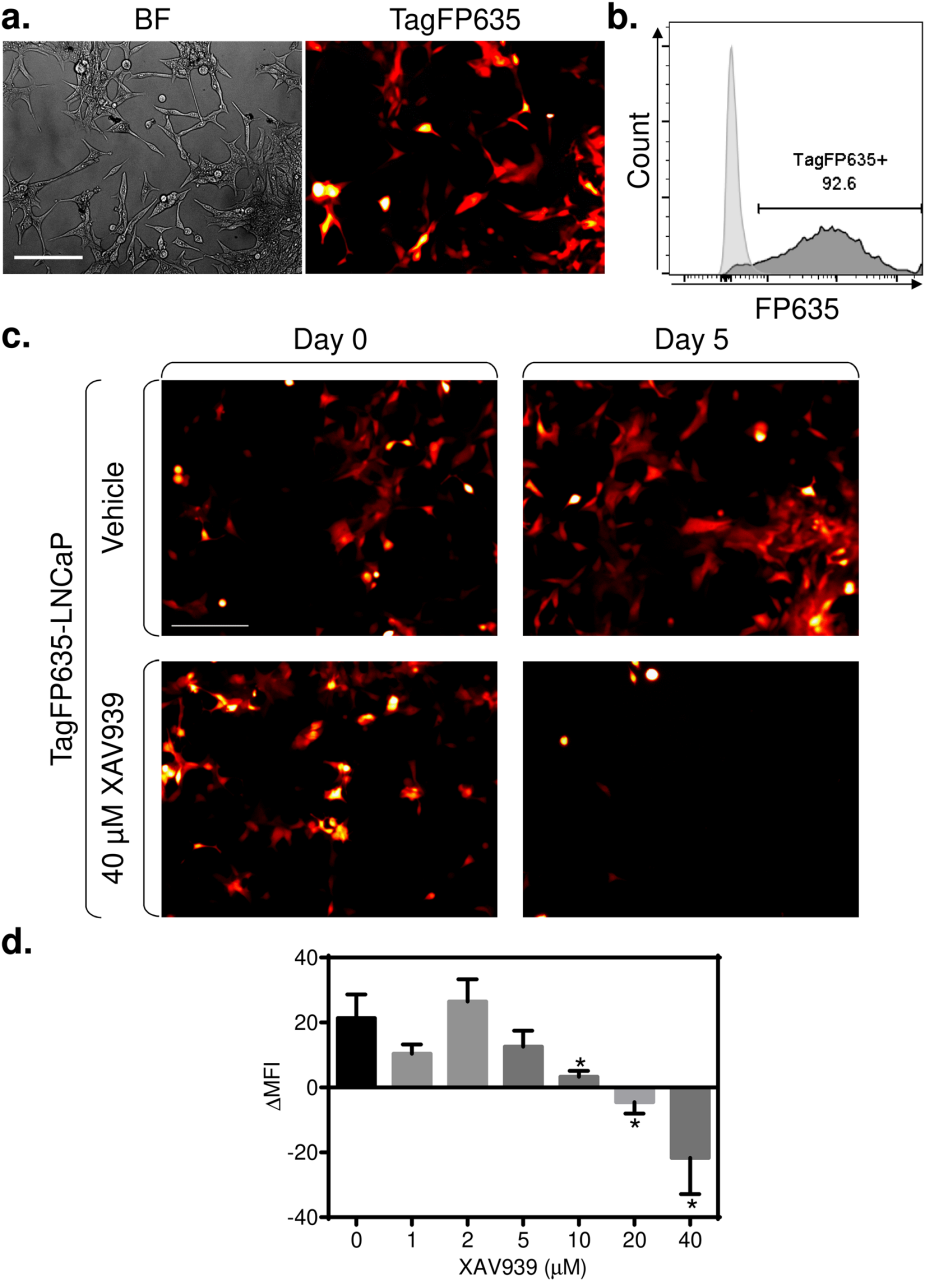
XAV939 (5 µM) did not impact the expansion of cultured TagFP635-LNCaP cells. (**a**) Bright field (BF) and TagFP635 fluorescence (TagFP635) images of cultured TagFP635-LNCaP cells at a 10 x magnification. (**b**) Flow cytometric analysis of live (DAPI^−^) TagFP635-LNCaP cells and their non-transformed counterpart (LNCaP) as a negative control. (**c**) TagFP635-LNCaP cells were cultured in the presence or absence (Vehicle) of 40 μM XAV939 for 2 days. Images of TagFP636 fluorescence in the culture were acquired before and after culturing. (**d**) TagFP635-LNCaP cells were cultured in the presence or absence of the indicated concentrations of XAV939 for 2 days. Images were acquired at a 10 x magnification. The mean fluorescence intensities (MFI) of the acquired images were calculated. Then, the difference between the MFIs before and after culturing (ΔMFI) was calculated and statistically evaluated by the Mann-Whitney test (LNCaP cells, *n* = 6 independent experiments). ^*^P < 0.05. The data are shown as the mean ± SEM. In **a** and **c**, the bar in the top left image represents 200 µm.

XAV939-mediated inhibition of Wnt/β-catenin signaling in cancer cells is often associated with inhibition of cell growth and/or induction of apoptosis^28–30^. To determine how XAV939 impacts the expansion of prostate cancer cells during culture, we stably transfected LNCaP cells^22^ with a red fluorescent protein (TagFP635) using a lentiviral system. Transfected cells were positively selected with puromycin. Selected cells were well detected in the RFP channel of the microscope (Fig. 1a), and over 90% of the cells in the culture were stably positive for fluorescence during subsequent culturing, as determined by flow cytometry (Fig. 1b). These cells were then cultured in the presence of increasing concentrations of XAV939, and cell expansion was determined according to the intensity of their fluorescence (Fig. 1c). As shown in Fig. 1d, expansion of TagFP635-LNCaP cells for 2 days was not affected by XAV939 at concentrations of up to 5 µM. At a concentration of 10 µM XAV939, expansion was reduced, and at 40 µM XAV939, the culture contracted, which indicated the cytotoxicity of XAV939 in LNCaP cells at this concentration. In the following experiments, we used 5 µM XAV939, which we found to be the maximal concentration that did not affect the expansion of LNCaP cells.

### Proliferation of BRPCa CD4^+^ and CD8^+^ lymphocytes is not impacted by 5 µM XAV939

**Figure 2:**
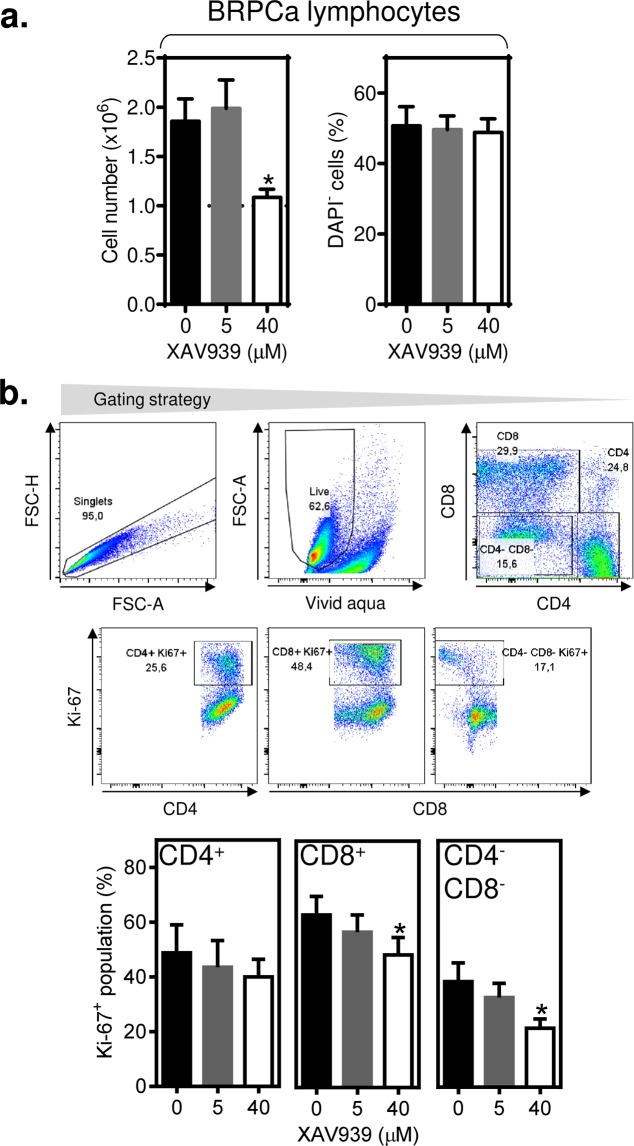
XAV939 (5 µM) does not impact the proliferation and viability of BRPCa lymphocytes. (**a**) BRPCa lymphocytes (1.0 × 10^6^ cells) were stimulated with 5 μg/ml phytohaemagglutinin (PHA) and cultured for 3 days, and the cell number (*left panel*) and cell viability (DAPI^−^ population, *right panel*) were determined. (**b**) The cells in (**a**) were stained with LIVE/DEAD Fixable Aqua Stain, fixed, permeabilized, and stained with CD4−, CD8− and Ki-67-specific antibodies, and the population of Ki-67^+^ in live (Aqua Stain^−^) single cells of CD4^+^, CD8^+^ and CD4^−^CD8^−^ lymphocytes was determined (gating strategy in first 2 rows) and evaluated (bottom panels). In (**a**) and (**b**), the differences between XAV939-untreated and treated BRPCa lymphocytes were statistically evaluated (Wilcoxon matched-pairs signed-ranks test, *n* = 7 patients). ^*^P < 0.05. The data are shown as the mean ± SEM.

In the following experiments, we tested how XAV939 impacts the expansion of BRPCa lymphocytes. We first tested how 3-day proliferation of phytohaemagglutinin (PHA)-stimulated BRPCa lymphocytes was affected by 5 µM XAV939. The LNCaP cell-toxic concentration of 40 µM XAV939 was used as a control. As shown in Fig. 2a, 5 µM XAV939 had no impact on the proliferation of PHA-stimulated cells. On the other hand, 40 µM XAV939 entirely abrogated this proliferation. This effect was, however, not associated with a decreased viability of the tested cells (Fig. 2a). We next analyzed how the proliferation of CD4^+^, CD8^+^, and CD4^−^CD8^−^ BRPCa lymphocytes was affected by XAV939 using a Ki-67 assay. As shown in Fig. 2b, 5 µM XAV939 had no inhibitory effect on the expression of Ki-67 in the tested lymphocytes. However, an inhibitory effect was observed with 40 µM XAV939 in CD8^+^ and CD4^-^CD8^-^ lymphocytes. Surprisingly, CD4^+^ lymphocytes were more refractory to inhibition even with this concentration of the compound, showing that the homeostasis of immune cell populations can be differentially affected by this Wnt/β-catenin signaling inhibitor. Collectively, these data showed that 5 µM XAV939 had no effect on the proliferation and viability of BRPCa lymphocytes.

### Significant inhibition of β-catenin translocation to the nucleus in LNCaP cells is attained with 5 µM XAV939

**Figure 3:**
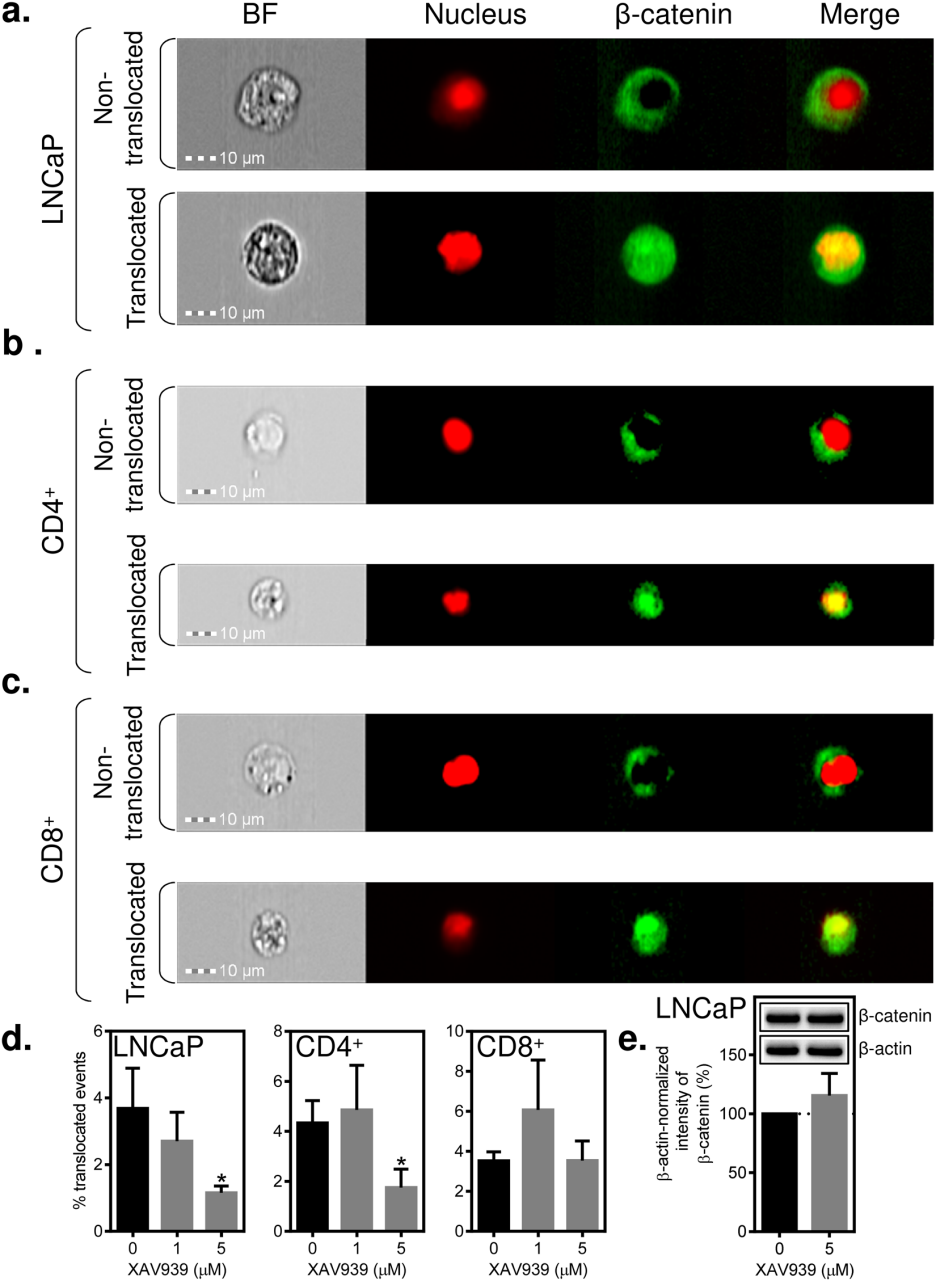
XAV939 (5 µM) inhibits β-catenin translocation to the nucleus in LNCaP cells and BRPCa CD4^+^ lymphocytes, but not in BRPCa CD8^+^ lymphocytes. (**a**–**c**) LNCaP cells and BRPCa lymphocytes were cultured in the presence of 5 µM XAV939 for 2 days. Lymphocytes were stained with CD4- and CD8-specific antibodies prior to fixation and permeabilization. Fixed and permeabilized cells were stained with a β-catenin-specific antibody and DRAQ5 to stain the nucleus. The stained LNCaP cells (**a**) and the stained and gated CD4^+^ (**b**) and CD8^+^ (**c**) lymphocytes were analyzed by an imaging flow cytometer, ImageStreamX MKII. Bright field (BF) and the fluorescent images of the nucleus (red, DRAQ5) and β-catenin (green) of gated single cells were collected at a 40 x magnification, and 1.0 × 10^5^ cells were acquired for each sample. Representative images are shown, and the bars in the left bottom corner of the bright field images represent 10 µm. (**d**) LNCaP cells and BRPCa lymphocytes were cultured in the presence of the indicated concentrations of XAV939 for 2 days and analyzed as in (**a**–**c)**. Nuclear localization of β-catenin was determined by a Pearson correlation, and statistical significance between the number of cells with β-catenin translocated to the nucleus in the vehicle- and XAV939-treated samples was evaluated by the Mann-Whitney test (LNCaP cells, *n* = 5–7 independent experiments) or Wilcoxon matched-pairs signed-ranks test (BRPCa lymphocytes, *n* = 5 patients). ^*^P < 0.05. (**e**) Immunoblot analysis of β-catenin in LNCaP cells treated or not for 2 days with 5 µM XAV939. The intensity of β-catenin was normalized to the intensity of β-actin, and the differences between the XAV939-treated and non-treated samples were statistically evaluated by the Mann-Whitney test (*n* = 3 independent experiments). ^*^P < 0.05. Representative images of immunoblots from the same gel are shown. The data in (**d**–**e**) are shown as the mean ± SEM.

The concentration of XAV939 that efficiently inhibits Wnt/β-catenin signaling in transformed cells varies^31^. To comparably test the inhibitory efficacy of XAV939 on the indices of signaling in both cancer cell and lymphocyte populations, we used imaging flow cytometry. We treated LNCaP cells for 2 days with XAV939 at concentrations that did not impact LNCaP expansion in culture (1 and 5 µM). Using imaging flow cytometry, we evaluated the inhibition of β-catenin translocation to the nucleus as a surrogate marker of Wnt/β-catenin signaling. As shown in Fig. 3a, LNCaP cells with β-catenin translocated or not translocated to the nucleus were well distinguished by imaging flow cytometry. We found that approximately 4% of the analyzed LNCaP cells had β-catenin translocated to the nucleus (Fig. 3d). This population, however, was significantly reduced in the presence of 5 µM XAV939 (Fig. 3d). This reduction was not, however, associated with a reduction of the total β-catenin in cells, as determined by immunoblotting (Fig. 3e). Collectively, these data showed that although 5 µM XAV939 was not able to affect LNCaP expansion in culture, it was able to inhibit β-catenin translocation to the nucleus without altering its total protein level in these cells.

### XAV939 (5 µM) inhibits β-catenin translocation to the nucleus in BRPCa CD4^+^ lymphocytes but not CD8^+^ lymphocytes

In the next array of experiments, we tested whether 5 µM XAV939 also inhibited β-catenin translocation to the nucleus in immune cells. We used BRPCa lymphocytes and evaluated the inhibition of β-catenin translocation to the nucleus with imaging flow cytometry in CD4^+^ and CD8^+^ lymphocytes. As shown in Fig. 3b,c, both CD4^+^ and CD8^+^ BRPCa lymphocytes with β-catenin translocated or not translocated to the nucleus were well distinguished by imaging flow cytometry. Similar to LNCaP cells, approximately 4% of the populations of CD4^+^ and CD8^+^ BRPCa lymphocytes were found to have β-catenin translocated to the nucleus (Fig. 3d). However, unlike LNCaP cells, 5 µM XAV939 reduced the β-catenin-translocated population only in CD4^+^ lymphocytes (Fig. 3d). No reduction was found in CD8^+^ lymphocytes (Fig. 3d). These data showed that although 5 µM XAV939 did not have an effect on the proliferation and viability of BRPCa lymphocytes, different subsets of lymphocytes can be differently sensitive to XAV939-mediated inhibition of β-catenin translocation to the nucleus.

### BRPCa lymphocytes preconditioned with 5 µM XAV939 accelerate the elimination of LNCaP cells

In the first part of the study, we found that 5 µM XAV939 had no impact on the expansion/proliferation or viability of LNCaP cells and BRPCa lymphocytes, but it was still able to inhibit β-catenin translocation to the nucleus in these cells or in their subset. Thus, this concentration of XAV939 did not affect the critical events that are necessary to evaluate immune cell-mediated elimination of cancer cells.

**Figure 4:**
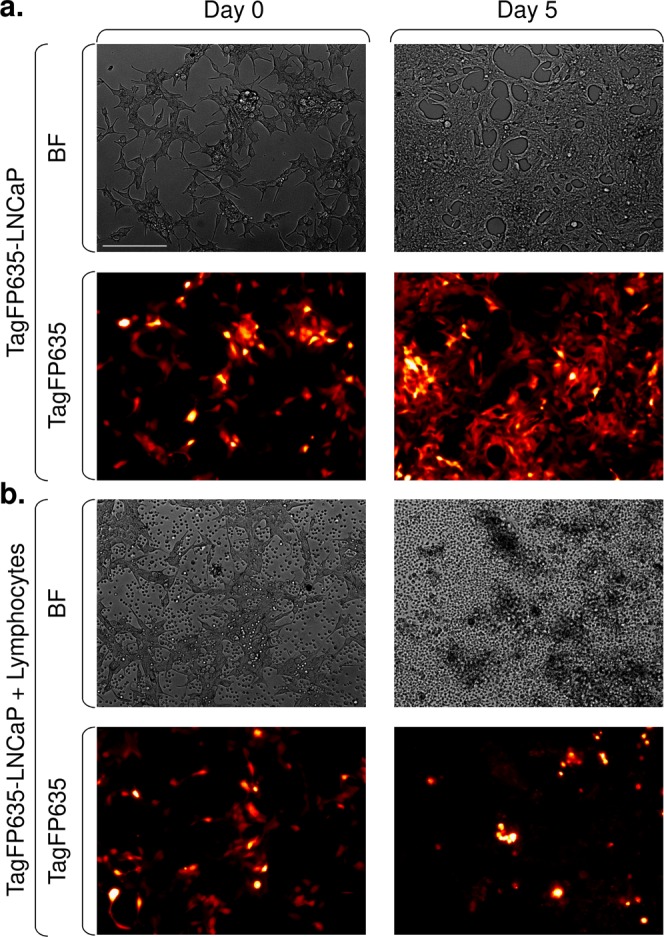
BRPCa lymphocytes eliminate TagFP635-LNCaP cells during coculturing. (**a**) TagFP635-LNCaP cells alone were cultured for 5 days, and bright field (BF) and TagFP635 fluorescence (TagFP635) images were acquired before and after culturing. (**b**) TagFP635-LNCaP cells were cocultured with BRPCa lymphocytes for 5 days, and images were acquired as in (**a**). Representative images of at least 3 independent cocultures at a 10 x magnification are shown. The bar in the top left image represents 200 µm.

**Figure 5:**
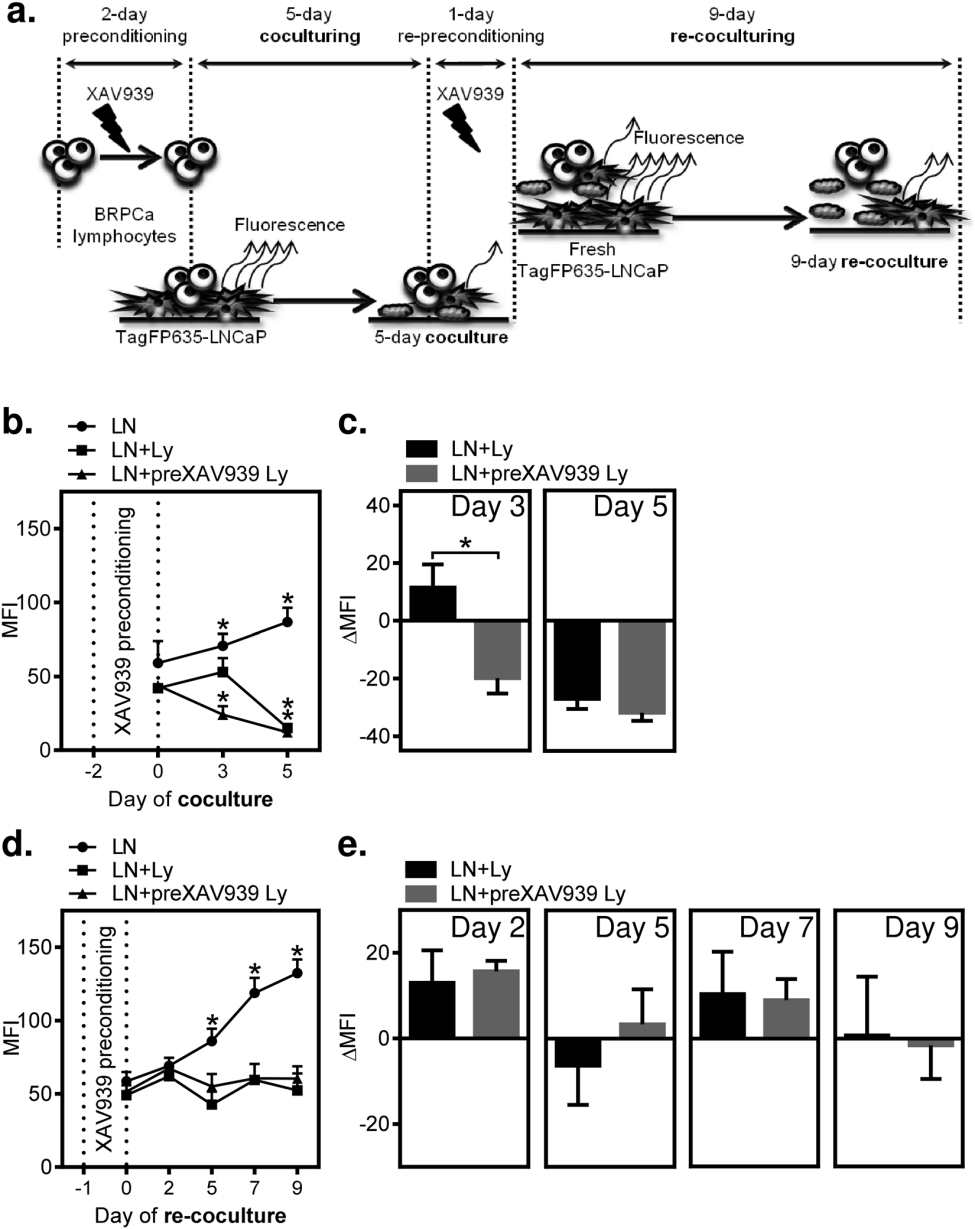
Preconditioning of BRPCa lymphocytes with 5 μM XAV939 accelerates the elimination of TagFP635-LNCaP cells during coculturing, but fails to counteract the acquired lymphocyte inability to eliminate fresh TagFP635-LNCaP cells during re-coculturing. (**a**) Schematic of the experiment. (**b**) BRPCa lymphocytes were (LN + preXAV939 Ly) or not (LN + Ly) preconditioned with 5 μM XAV939 for 2 days and then cocultured with TagFP635-LNCaP cells for 5 days. TagFP635-LNCaP cells cultured without lymphocytes (LN) were used as a control. Images of TagFP636 fluorescence in the coculture were acquired on days 0, 3 and 5 of coculturing, and the mean fluorescence intensity (MFI) of the acquired images was calculated. The differences in the MFIs of each individual sample at day 0 and the following days of coculturing (days 3 and 5) were statistically evaluated (Mann-Whitney test; LNCaP cells, *n* = 6 independent experiments, BRPCa lymphocytes, *n* = 6 patients). (**c**) The differences between the MFIs before coculturing (day 0) and at each individual day of coculturing (day 3 and 5) (ΔMFIs) were calculated for the LN + Ly and LN + preXAV939 samples and statistically evaluated (Wilcoxon matched-pairs signed-ranks test, *n* = 6 patients). (**d**) Five-day cocultured cells in (**b**) were re-preconditioned (LN + preXAV939 Ly) or not (LN + Ly) by 1-day of coculture in the presence of 5 μM XAV939 and then transferred to fresh TagFP635-LNCaP cells and re-cocultured for 9 days. Fresh TagFP635-LNCaP cells cultured without lymphocytes (LN) were used as a control. Images of TagFP636 fluorescence in the re-coculture were acquired on days 2, 5, 7 and 9 of re-coculturing, and the MFIs of the acquired images were calculated. The differences in the MFIs of each individual sample at day 0 and the following days of re-coculturing (days 2, 5, 7 and 9) were statistically evaluated (Mann-Whitney test; LNCaP cells, *n* = 6 independent experiments, BRPCa lymphocytes, *n* = 6 patients). (**e**) The differences between the MFIs before re-coculturing (day 0) and at each individual day of re-coculturing (day 2, 5, 7 and 9) (ΔMFIs) were calculated for the LN + Ly and LN + preXAV939 Ly samples and statistically evaluated (Wilcoxon matched-pairs signed-ranks test, *n* = 6 patients). In (**b**–**e**) ^*^P < 0.05, the data are shown as the mean ± SEM.

Thus, in the next set of experiments, we performed extended coculture studies of TagFP635-LNCaP cells cocultured with BRPCa lymphocytes in which 5 µM XAV939 was used to affect both cell types individually or simultaneously. We first tested how the expansion of TagFP635-LNCaP cells was affected by coculturing these cells with BRPCa lymphocytes. As shown in Fig. 4, the presence of BRPCa lymphocytes in coculture with TagFP635-LNCaP cells did not interfere with the TagFP635 fluorescent signal. When TagFP635-LNCaP cells were cultured alone, both the content of cells and fluorescence in the culture increased (Fig. 4a). However, when TagFP635-LNCaP cells were cocultured with BRPCa lymphocytes, TagFP635-LNCaP cells were eliminated and the fluorescent signal nearly completely disappeared (Fig. 4b). Similar results were observed when BRPCa lymphocytes were preconditioned for 2 days with 5 µM XAV939 (Suppl. Fig. 1). No fluorescent signal was detected when BRPCa lymphocytes were cultured alone (data not shown). This result showed that this coculture system was able to specifically monitor the impact of immune cells on the expansion of cancer cells. Using this system, we first tested how preconditioning BRPCa lymphocytes with 5 µM XAV939 for 2 days affected their performance in the elimination of TagFP635-LNCaP cells in the coculture system (Fig. 5a). As shown in Fig. 5b, TagFP635-LNCaP cells expanded after 5 days of coculturing. On the other hand, 5-days of co-culture with BRPCa lymphocytes led to the elimination of TagFP635-LNCaP cells from the culture regardless of whether the BRPCa lymphocytes were preconditioned with XAV939. However, XAV939 preconditioning significantly accelerated this elimination because at day 3 of coculture, BRPCa lymphocytes only abrogated the expansion of TagFP635-LNCaP cells, but their XAV939-preconditioned counterparts were already eliminating them from culture (Fig. 5c). To determine whether the faster elimination of TagFP635-LNCaP cells by XAV939-preconditioned BRPCa lymphocytes was also preserved after extended coculturing, we harvested BRPCa lymphocytes after 5 days of coculture, re-preconditioned them for 1 day with 5 µM XAV939, transferred them to a fresh culture of TagFP635-LNCaP cells and re-cocultured them for 9 days. As shown in Fig. 5d,e, BRPCa lymphocytes were still able to abrogate the expansion of fresh TagFP635-LNCaP cells in the 9 days of re-coculturing. However, BRPCa lymphocytes were no longer able to eliminate TagFP635-LNCaP cells regardless of re-preconditioning with XAV939 prior to re-coculturing. These data showed that the faster elimination of TagFP635-LNCaP cells by XAV939-preconditioned BRPCa lymphocytes was not only no longer observed during re-coculturing but that BRPCa lymphocytes were also no longer able to eliminate TagFP635-LNCaP cells during this process.

### XAV939 (5 µM) is critical for preserving BRPCa lymphocyte-mediated elimination of LNCaP cells during re-coculturing

**Figure 6:**
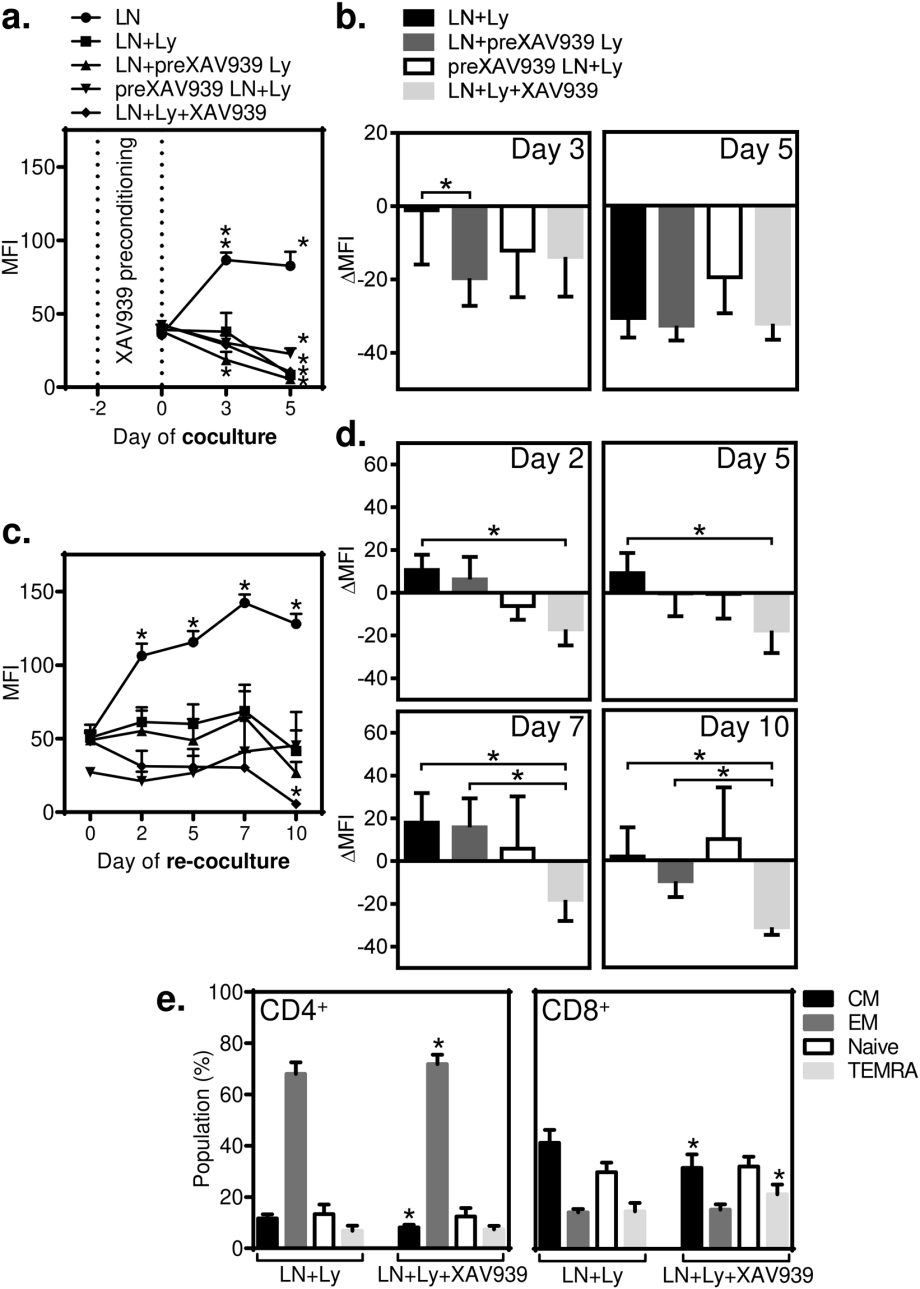
Acquired inability of BRPCa lymphocytes to eliminate TagFP635-LNCaP cells during re-coculturing is counteracted by coculturing and re-coculturing in the presence of 5 µM XAV939. (**a**) BRPCa lymphocytes were preconditioned with 5 μM XAV939 for 2 days (LN + preXAV939 Ly) or not (LN + Ly and preXAV939 LN + Ly), and TagFP635-LNCaP cells were preconditioned with 5 μM XAV939 for 2 days (preXAV939 LN + Ly) or not (LN and LN + preXAV939 Ly). The cells were then cocultured for 5 days. TagFP635-LNCaP cells cultured without lymphocytes (LN) were used as a control, and TagFP635-LNCaP cells and BRPCa lymphocytes that were not preconditioned with 5 μM XAV939 but cocultured in its presence from day 0 (LN + Ly + XAV939) were used to determine the impact of XAV939 supplementation during coculturing. Images of TagFP636 fluorescence in the coculture were acquired on days 0, 3 and 5 of coculturing, and the mean fluorescence intensity (MFI) of the acquired images was calculated. The differences in the MFIs of each individual sample at day 0 and the following days of coculturing (days 3 and 5) were statistically evaluated (Mann-Whitney test; LNCaP cells, *n* = 6 independent experiments, BRPCa lymphocytes, *n* = 12 patients). (**b**) The differences between the MFIs before coculturing (day 0) and at each individual day of coculturing (day 3 and 5) (ΔMFIs) were calculated for the LN + Ly, LN + preXAV939, preXAV939 LN + Ly, and LN + Ly + XAV939 samples and statistically evaluated (Wilcoxon matched-pairs signed-ranks test, *n* = 12 patients). (**c**) Five-day cocultured cells were next transferred to fresh TagFP635-LNCaP cells and re-cocultured for 10 days. Fresh TagFP635-LNCaP cells cultured without lymphocytes (LN) were used as a control. Images of TagFP636 fluorescence in the re-coculture were acquired on days 2, 5, 7 and 10 of re-coculturing, and the MFIs of the acquired images were calculated. The differences in the MFIs of each individual sample at day 0 and the following days of re-coculturing (day 2, 5, 7 and 10) were statistically evaluated (Mann-Whitney test; LNCaP cells, *n* = 6 independent experiments, BRPCa lymphocytes, *n* = 12 patients). (**d**) The differences between the MFIs before the re-coculturing (day 0) and at each individual day of re-coculturing (day 2, 5, 7 and 10) (ΔMFIs) were calculated for the LN + Ly, LN + preXAV939, preXAV939 LN + Ly, and LN + Ly + XAV939 samples and statistically evaluated (Wilcoxon matched-pairs signed-ranks test, *n* = 12 patients). ^*^P < 0.05. (**e**) The BRPCa T lymphocyte phenotype was determined before and after 15-day co- and re-coculturing in (**a**,**b**) and (**c**,**d**). The population of live cells was gated as DAPI-negative cells, and the T lymphocyte population was gated as CD3^+^ cells. Gated CD4^+^ or CD8^+^ T lymphocytes with the phenotype of central memory (CM) were CD45RO^+^CCR7^+^, effector memory CD45RO^+^CCR7^-^, naive CD45RO^-^CCR7^+^, and terminally differentiated (TEMRA) CD45RO^-^CCR7^-^. The differences in the content of each T lymphocyte population between the LN + Ly and LN + Ly + XAV939 samples were statistically evaluated (Wilcoxon matched-pairs signed-ranks test, *n* = 12 patients). In (**a**–**e**) ^*^P < 0.05, the data are shown as the mean ± SEM.

The coculture experiments showed that after 5 days, TagFP635-LNCaP cells were eliminated from the culture in all the tested samples but this elimination was no longer observed after re-coculturing with fresh cancer cells regardless of lymphocyte preconditioning with XAV939. Therefore, we next tested whether preconditioning TagFP635-LNCaP cells with XAV939 or coculturing/re-coculturing them in the presence of 5 µM XAV939 was able to counteract this acquired lymphocyte disability. As shown in Fig. 6a–d, these interventions had no benefit on cancer cell elimination during the initial 5 day coculture. However, during re-coculturing with fresh TagFP635-LNCaP cells, only the cells cocultured and then re-cocultured in the presence of 5 µM XAV939 were able to eliminate cancer cells during re-coculturing. In summary, these data demonstrated that the presence of 5 µM XAV939 during co- and re-coculturing effectively counteracted the acquired lymphocyte disability to eliminate fresh TagFP635-LNCaP cells during re-coculturing.

### XAV939 (5 µM) during cancer cell elimination minimally affects T lymphocyte differentiation

T lymphocytes differentiate when they encounter antigens, and this differentiation is affected by Wnt/β-catenin signaling in T lymphocytes. Altered differentiation of T lymphocytes affects the resultant proportion of naive, effector memory (EM), central memory (CM) and terminally differentiated (TEMRA) T lymphocytes^32^. Because our initial analysis revealed that 2-day preconditioning of BRPCa lymphocytes with 5 µM XAV939 inhibited β-catenin translocation to the nucleus in their CD4^+^ subset, we next tested whether BRPCa T lymphocyte differentiation after 15-day co-/re-coculturing with TagFP635-LNCaP cells was affected by the presence of 5 µM XAV939. As shown in Fig. 6e, 5 µM XAV939 minimally affected the proportions of naive, effector memory, central memory, and terminally differentiated T lymphocytes even after cancer cell elimination via 15-day co-/re-coculturing.

### XAV939 (5 µM) impacts BRPCa lymphocyte-mediated elimination of PC-3 cells

Our findings in LNCaP cells prompted us to determine whether 5 µM XAV939 also impacted BRPCa lymphocyte-mediated elimination in other prostate cancer cells. Therefore, we transfected a PC-3 prostate cancer cell line^23^ with a red fluorescent protein (TagFP635) using a lentiviral system. Transfected PC-3 cells were, similar to transfected LNCaP cells, over 90% stably positive for fluorescence during culturing, and their expansion was not affected by 5 µM XAV939 (Suppl. Fig. 2). Similar to LNCaP cells, BRPCa lymphocytes preconditioned with 5 µM XAV939 accelerated PC-3 elimination (Suppl. Fig. 3) and the presence of 5 µM XAV939 was critical to the preservation of lymphocyte-mediated PC-3 cell elimination during re-coculturing (Suppl. Fig. 4).

## Discussion

In this work, we describe a novel mode of action of the Wnt/β-catenin signaling inhibitor XAV939 on immune cell-mediated elimination of PCa cells. We used an *in vitro* coculture system and the Wnt/β-catenin signaling inhibitor XAV939 at a concentration that inhibited β-catenin translocation to the nucleus in cancer cells and a subset of immune cells without affecting their proliferation, viability, and differentiation. This coculture system allowed us to determine the contribution of the inhibited component of Wnt/β-catenin signaling, β-catenin translocation to the nucleus, in both cell types to the process of lymphocyte-mediated elimination of PCa cells. We found that BRPCa lymphocytes initially eliminated TagFP635-LNCaP cells during coculturing and that this process was accelerated by preconditioning BRPCa lymphocytes with XAV939. Re-coculturing these lymphocytes with fresh TagFP635-LNCaP cells, however, revealed that during the initial coculture, lymphocytes acquired an inability to further eliminate cancer cells and that this acquired inability was only counteracted when the cells were cocultured and re-cocultured with XAV939. Comparable results were also obtained when TagFP635-PC-3 cells were used.

XAV939 is a tankyrase inhibitor that targets Wnt/β-catenin signaling^33^. Inhibitors of Wnt/β-catenin signaling have been tested in multiple clinical studies^15^. The first mode of action of these inhibitors is an increase in Wnt/β-catenin signaling in multiple cancer cells, and its inhibition can lead to decreased proliferation^28,29^ or even compromised viability of these cells^30^. The second mode of action of these inhibitors is that inhibition of Wnt/β-catenin signaling in cancer cells can increase their radiosensitivity, chemosensitivity or hormonal sensitivity^34–36^. Which of these two modes of action is dominant is concentration dependent. In LNCaP cells, 10 µM XAV939 was found to only partially inhibit their growth^36^, whereas 1 µM XAV939 was able to radiosensitize them^37^. Our results are consistent with these prior studies, as we found that 40 µM XAV939 was cytotoxic to TagFP635-LNCaP cells, 10 µM XAV939 partially inhibited their expansion and 5 µM XAV939 had no effect on their expansion. However, unlike chemosensitization, 5 µM XAV939, which was still able to inhibit β-catenin translocation to the nucleus in LNCaP cells, did not condition these cells to faster elimination by BRPCa lymphocytes.

Wnt/β-catenin signaling inhibitors, in *in vivo* studies, have shown benefits in treating different cancers^15^. It is believed that these inhibitors increase treatment efficacy primarily by increasing the sensitivity of cancer cells to the immune system by decreasing cancer cell stemness^38^. On the other hand, the increased sensitivity to the immune system can also be caused by the inhibition of Wnt/β-catenin signaling in immune cells, which promotes their immunostimulatory and anti-tolerogenic activity^18,19,39–42^. Our data support a prevalent role of Wnt/β-catenin signaling in immune cells at early stages of the coculture experiments in which XAV939 preconditioning of BRPCa lymphocytes but not of TagFP635-LNCaP cells increased lymphocyte-mediated elimination of cancer cells. This XAV939-mediated increase in performance, however, did not provide lymphocytes with any advantage after they were re-cocultured with fresh TagFP635-LNCaP cells; during re-coculturing, lymphocytes were unable to eliminate fresh cancer cells. This inability to respond to cancer cells was acquired during the initial coculture with TagFP635-LNCaP cells. LNCaP cells have upregulated Wnt/β-catenin signaling^43^, and upregulated Wnt/β-catenin signaling in cancer cells can suppress immune cell responses^12,13^. Inhibition of this suppression is the third known mode of action of Wnt/β-catenin signaling inhibitors that contributes to their treatment efficacy^12^. Our data showed that the acquired inability of lymphocytes to further eliminate fresh cancer cells was only counteracted when coculturing and re-coculturing were performed in the presence of XAV939. Because no such effect was observed when lymphocytes alone were exposed to XAV939 during preconditioning and re-preconditioning, these data therefore suggest that the sustained inhibition of β-catenin translocation to the nucleus in LNCaP cells elicited by XAV939 was the prevailing mechanism that prevented the acquisition of the lymphocyte disability.

Many approaches in immunotherapy of PCa have shown considerable efficacy in promoting PCa-targeted immune responses^44,45^. These responses are often associated with increased infiltration of immune cells into tumors, increased activation and/or increased numbers of tumor-associated antigen-specific lymphocytes^46–48^. However, despite these often robust immune responses, these immunotherapies have not yet adequately led to the expected therapeutic efficacy. Therefore, a single strategy for promoting immune responses against PCa cells is not sufficient to generate a substantial therapeutic impact; therefore, these approaches need to be combined with other treatment modalities. Inhibition of Wnt/β-catenin signaling has already been shown to be able to overcome resistance to PCa chemotherapy^21^. In this study, the use of the Wnt/β-catenin signaling inhibitor XAV939 in both PCa cells and patients’ lymphocytes was shown to potentiate the elimination of PCa cells *in vitro*. This potentiation can then improve the efficacy of PCa immunotherapy, where the therapeutic impact is largely delivered through lymphocyte-mediated elimination of cancer cells.

## Supplementary information

Supplementary Material
