# Supplementary material for: The WNT/β-catenin signaling inhibitor XAV939 enhances the elimination of LNCaP and PC-3 prostate cancer cells by prostate cancer patient lymphocytes in vitro
Source: Sci Rep. 2019 Mar 18;9:4761. doi: 10.1038/s41598-019-41182-5 (PMC6423115; doi:10.1038/s41598-019-41182-5)
Supplement: Supplementary file 1 — Supplementary Material [file 41598_2019_41182_MOESM1_ESM.pdf]

The WNT/β-catenin signaling inhibitor XAV939 enhances the elimination of LNCaP and PC-3 prostate cancer cells by prostate cancer patient lymphocytes *in vitro*

Dmitry Stakheev, Pavla Taborska, Zuzana Strizova, Michal Podrazil, Jirina Bartunkova, Daniel Smrz

Supplementary Figure Legends

Suppl. Fig. 1 BRPCa lymphocytes eliminate TagFP635-LNCaP cells during coculturing. TagFP635-LNCaP cells were cocultured for 5 days with BRPCa lymphocytes that were preconditioned with 5μM XAV939 for 2 days. Bright field (BF) and TagFP635 fluorescence (TagFP635) images were acquired before and after culturing. Representative images of at least 3 independent cocultures at a 10 x magnification are shown. The bar in the top left image represents 200 μm.

Suppl. Fig. 2 XAV939 (5 μM) does not impact the expansion of cultured TagFP635-PC-3 cells. (**a**) Bright field (BF) and TagFP635 fluorescence (TagFP635) images of cultured TagFP635-PC-3 cells at a 10 x magnification. (**b**) Flow cytometric analysis of live ($DAPI^-$) TagFP635-PC-3 cells and their non-transformed counterpart (PC-3) as a negative control. (**c**) TagFP635-PC-3 cells were cultured in the presence or absence of 5 μM XAV939 for 5 days. Images were acquired at a 10 x magnification. The mean fluorescence intensity (MFI) of the acquired images was calculated, and the difference between the MFIs before and after culturing (ΔMFI) was calculated and statistically evaluated by the Mann-Whitney test (*n*=5 independent experiments). *P<0.05. The data are shown as the mean±SEM.

Suppl. Fig. 3 Preconditioning BRPCa lymphocytes with 5 μM XAV939 accelerates the elimination of TagFP635-PC-3 cells during coculturing. (**a**) BRPCa lymphocytes were preconditioned (PC-3+preXAV939 Ly) or not (PC-3+Ly) with 5 μM XAV939 for 2 days

and then cocultured with TagFP635-PC-3 cells for 5 days. TagFP635-PC-3 cells cultured without lymphocytes (PC-3) were used as a control. TagFP635-PC-3 cells and BRPCa lymphocytes that were not preconditioned with 5μM XAV939 but cocultured in its presence from day 0 (PC-3+Ly+XAV939) were used to determine the impact of XAV939 supplementation during coculturing. Images of TagFP636 fluorescence in the coculture were acquired on days 0, 3 and 5 of coculturing, and the mean fluorescence intensity (MFI) of the acquired images was calculated. The differences in the MFIs of each individual sample at day 0 and the following days of coculturing (days 3 and 5) were statistically evaluated (Mann-Whitney test; PC-3 cells, *n*=5 independent experiments, BRPCa lymphocytes, *n*=6 patients). (**b**) The differences between the MFIs before coculturing (day 0) and at each individual day of coculturing (day 3 and 5) (ΔMFIs) were calculated for the PC-3+Ly and PC-3+preXAV939 Ly and PC-3+Ly+XAV939 samples and statistically evaluated (Wilcoxon matched-pairs signed-ranks test, *n*=6 patients). The data in (**a**–**b**) are shown as the mean-SEM.

Suppl. Fig. 4 Acquired inability of BRPCa lymphocytes to eliminate TagFP635-PC-3 cells during re-coculturing is counteracted by coculturing and re-coculturing in the presence of 5 μM XAV939. (**a**) The 5-day cocultured cells in Suppl. Fig. 3 were then transferred to fresh TagFP635-PC-3 cells and re-cocultured for 10 days. Fresh TagFP635-PC-3 cells cultured without lymphocytes (PC-3) were used as a control. Images of TagFP636 fluorescence in the re-coculture were acquired on days 2, 5, 7 and 10 of re-coculturing, and the MFIs of the acquired images were calculated. The differences in the MFIs of each individual sample at day 0 and the following days of re-

coculturing (day 2, 5, 7 and 10) were statistically evaluated (Mann-Whitney test; PC-3 cells, *n*=5 independent experiments, BRPCa lymphocytes, *n*=6 patients). (**d**) The differences between the MFIs before re-coculturing (day 0) and at each individual day of re-coculturing (day 2, 5, 7 and 10) (ΔMFIs) were calculated for the PC-3+Ly, PC-3+preXAV939 Ly, and PC-3+Ly+XAV939 samples and statistically evaluated (Wilcoxon matched-pairs signed-ranks test, *n*=6 patients). *P<0.05. The data in (**a**–**b**) are shown as the mean±SEM.

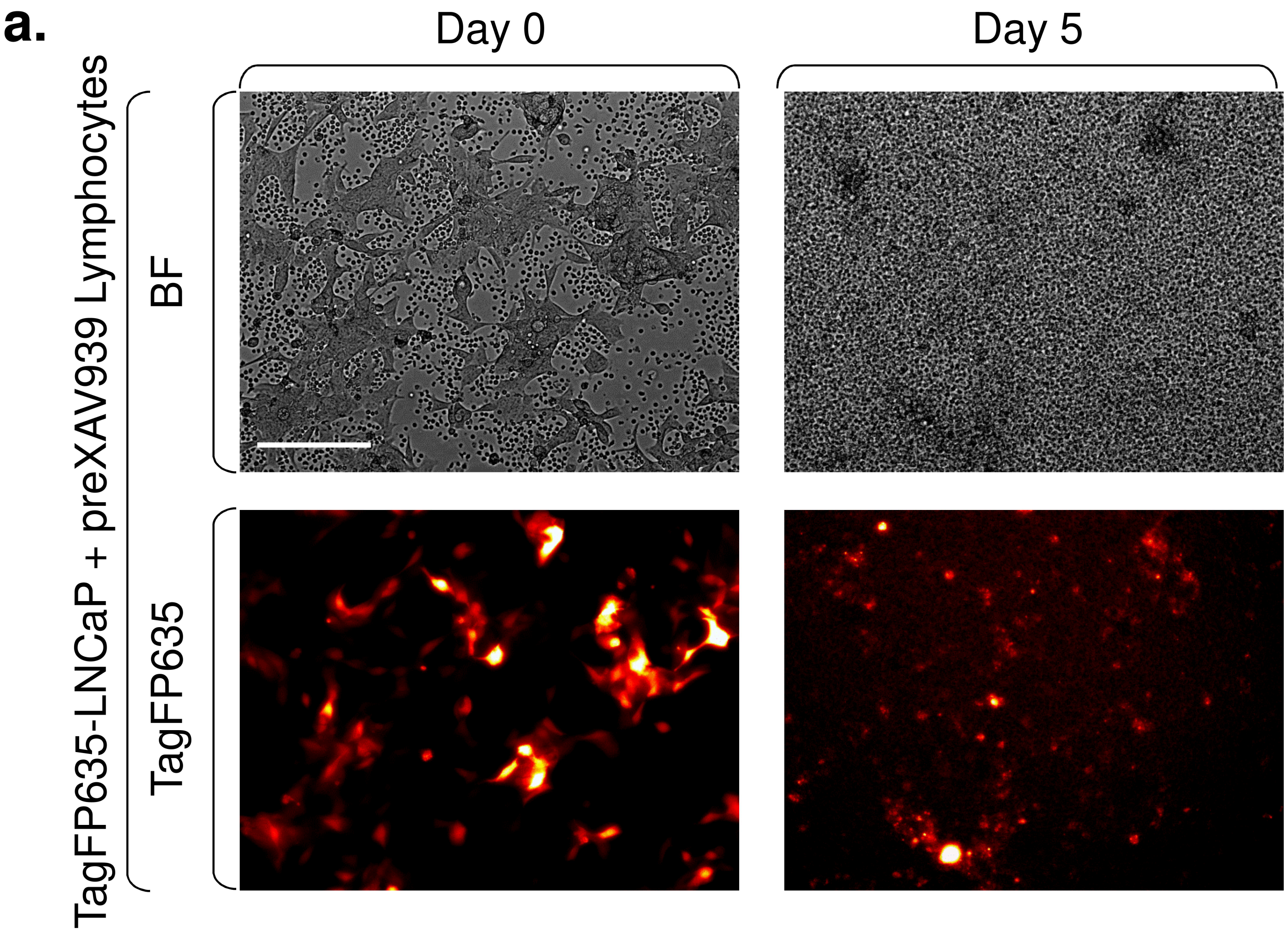

a.
Day 0
Day 5
TagFP635-LNCaP + preXAV939 Lymphocytes
BF
TagFP635

Suppl. Fig. 1

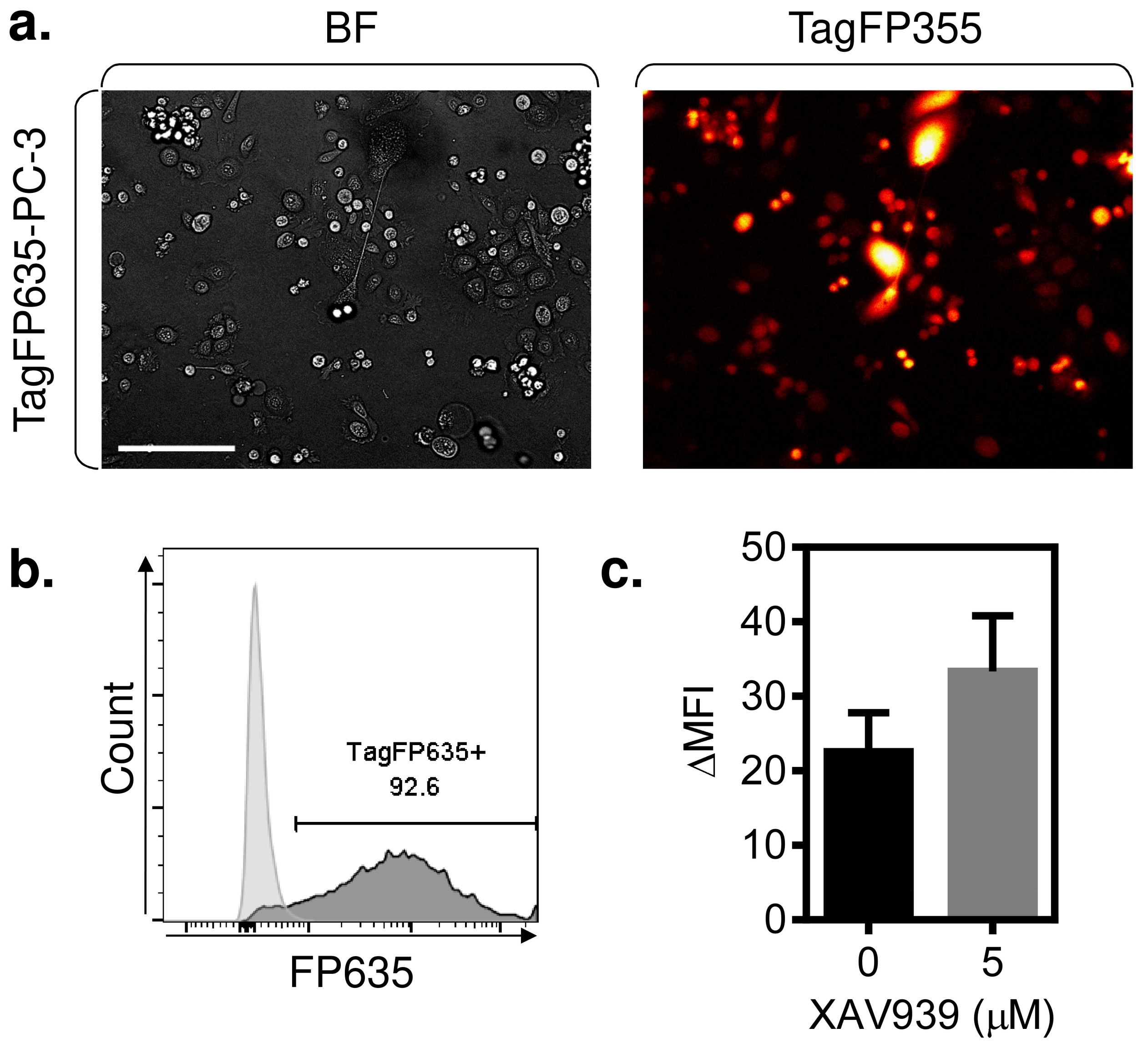

a.
BF
TagFP355
TagFP635-PC-3
b.
Count
TagFP635+
92.6
FP635
c.
50
40
30
20
10
0
ΔMFI
0
5
XAV939 (μM)

Suppl. Fig. 2

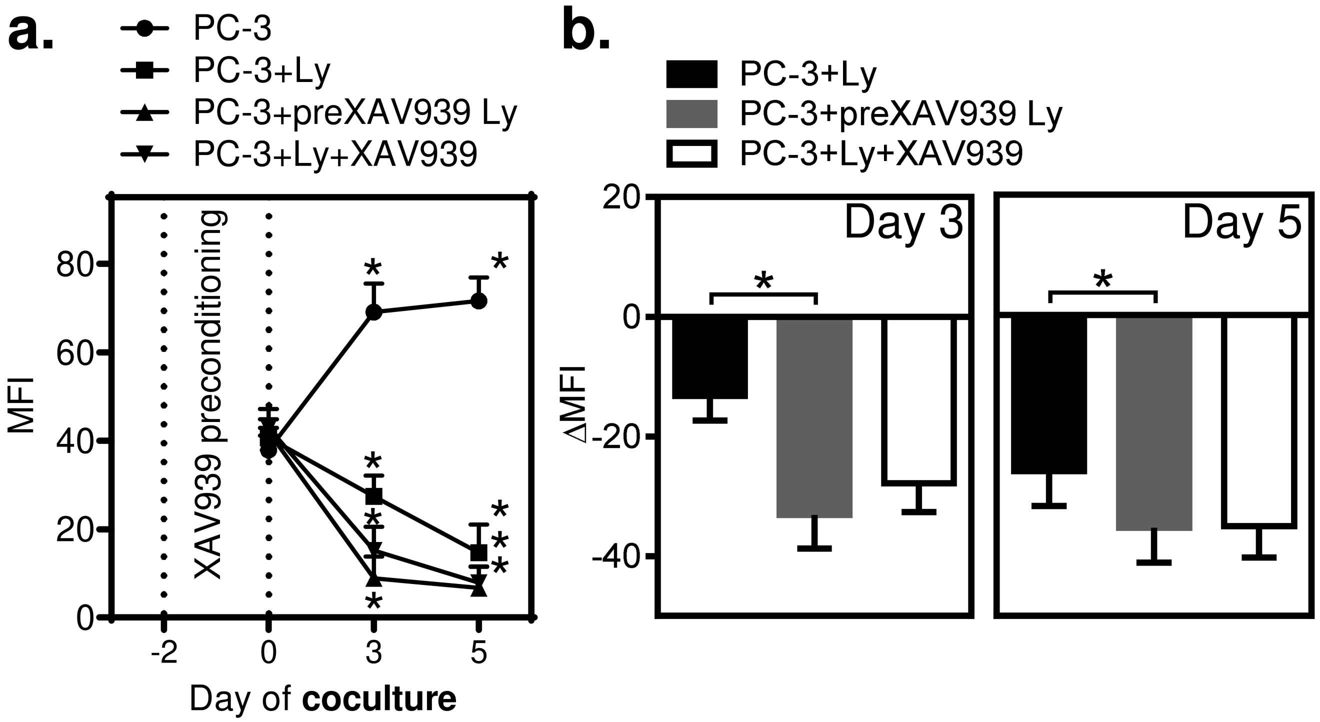
a.
PC-3
PC-3+Ly
PC-3+preXAV939 Ly
PC-3+Ly+XAV939
MFI
80
60
40
20
0
XAV939 preconditioning
*
-2
0
3
5
Day of **coculture**
b.
PC-3+Ly
PC-3+preXAV939 Ly
PC-3+Ly+XAV939
20
0
-20
-40
ΔMFI
Day 3
Day 5

Suppl. Fig. 3

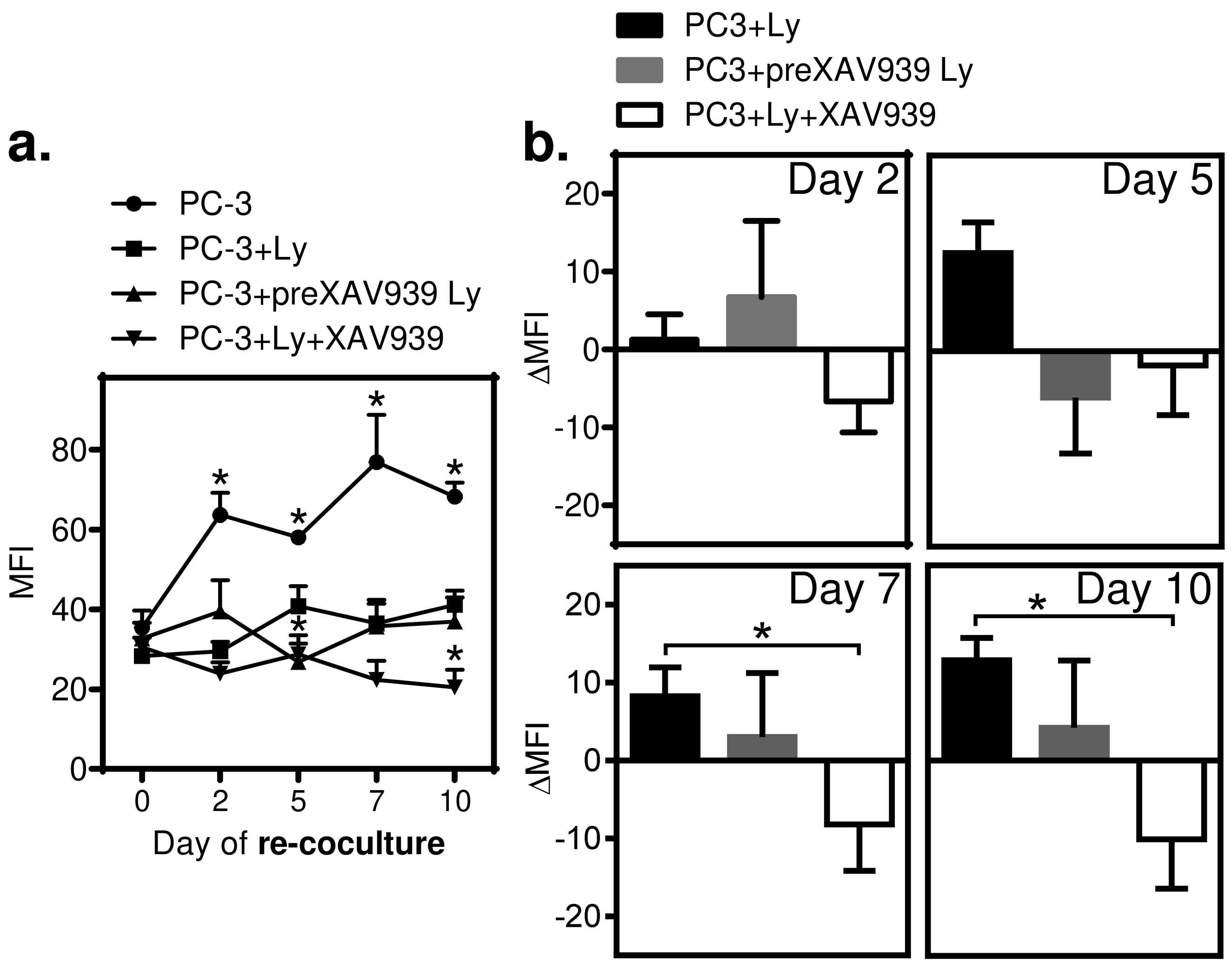

a.
PC-3
PC-3+Ly
PC-3+preXAV939 Ly
PC-3+Ly+XAV939
MFI
Day of **re-coculture**
b.
PC3+Ly
PC3+preXAV939 Ly
PC3+Ly+XAV939
Day 2
Day 5
Day 7
Day 10
ΔMFI

Suppl. Fig. 4
